# Identifying mental health outcomes and evidence-based psychological interventions for supporting pediatric gunshot wound patients: A systematic review and proposed conceptual model

**DOI:** 10.1186/s12887-024-04878-w

**Published:** 2024-06-18

**Authors:** Molly E. Hale, Kahyah Pinkman, Alexis M. Quinoy, Kindell R. Schoffner

**Affiliations:** 1 Department of Psychology, University of Georgia, Athens, GA USA; 2 Department of Neuropsychology and Rehabilitation Services, Children’s Healthcare of Atlanta, Atlanta, GA USA; 3 Department of Rehabilitation Medicine, Emory School of Medicine, Atlanta, GA USA

**Keywords:** Gunshot wound, Pediatrics, Mental health outcomes, Evidence-based psychological intervention

## Abstract

**Background:**

Accidental and assault gunshot wounds (GSWs) are the second leading cause of injury in the United States for youth ages 1- to 17-years-old, resulting in significant negative effects on pediatric patients’ mental health functioning. Despite the critical implications of GSWs, there has yet to be a systematic review synthesizing trends in mental health outcomes for pediatric patients; a gap the present review fills. Additionally, this review identifies evidence-based psychological interventions shown to be effective in the treatment of subclinical symptoms of psychological disorders in the general population.

**Methods:**

A comprehensive search was conducted using five databases: American Psychological Association (APA) PsycInfo, APA PsycArticles, Cumulative Index to Nursing and Allied Health Literature (CINAHL), Education Resource Information Center (ERIC), and Medical Literature Analysis and Retrieval Systems Online (MEDLINE). Twenty-two articles met inclusion criteria.

**Results:**

Findings suggest pediatric GSW patients are at a significantly elevated risk for mental health disorders when compared to other- (e.g., motor vehicle collision) and non-injured youth. Disorders include post-traumatic stress, disruptive behavior, anxiety, depression, and substance use. Hospital-based violence intervention programs, cultivating supportive relationships with adults in one’s community, and trauma-focused outpatient services were identified as effective interventions for treating subclinical psychological symptoms.

**Conclusions:**

Depicted in the proposed conceptual model, the present study delineates a direct association between pediatric GSWs and subsequent onset of mental health disorders. This relation is buffered by evidence-based psychological interventions targeting subclinical symptoms. Results suggest brief psychological interventions can help treat mental health challenges, minimizing risk for significant long-term concerns. Cultural adaptations to enhance the utility and accessibility of interventions for all patients are recommended.

**Supplementary Information:**

The online version contains supplementary material available at 10.1186/s12887-024-04878-w.

In 2019, accidental and assault gunshot wounds (GSWs) became the leading cause of death and the second highest cause of injury in youth ages 1- to 17-years-old in the United States [1]. Approximately 8,400 youth are impacted by GSWs annually: 2,200 die and 6,200 are left to cope with life-altering physical and mental health challenges [1, 2]. Accidental and assault GSWs comprise 83% of all pediatric firearm injuries and disproportionately effect Black youth. Comparatively, self-injurious and suicide-related GSWs make up only 14% of pediatric GSW injuries and are more common in White youth [3]. Unlike self-injurious and suicidal GSWs, accidental and assault injuries do not necessarily imply prior mental health challenges for pediatric patients [4, 5]. However, accidental and assault GSWs pose significant risk for the development of subclinical symptoms of psychological disorders that, when left untreated, often develop into anxiety, depressive, disruptive behavior, substance use, and post-traumatic stress disorders [4, 5]. Thus, treating subclinical symptoms of psychological disorders following a GSW is imperative to improve the mental health outcomes for this population. Despite the significant psychological impact that accidental and assault GSWs have, there has yet to be a rigorous examination of the literature regarding relations between GSWs and mental health outcomes while identifying evidence-based treatments for treating subclinical symptoms of psychological disorders.

In 1996, the Dickey Amendment was ratified by Congress in response to gun control debates. The amendment largely prohibited the allocation of federal funds towards gun control research [6]. In 2018, Congress modified the amendment by allotting $25 million towards research on GSW outcomes. Since the amendment, the majority of GSW research has investigated outcomes in adults. However, research has begun to examine mental health outcomes for pediatric patients [6–9]. Compared to adults, pediatric GSW patients differ in their clinical presentation and long-term psychological adjustment following injury and mental health outcomes are typically obtained through medical records [10, 11]. Given data on psychological adjustment for pediatric patients largely began in 2018 [6], a comprehensive synthesis of mental health outcomes following accidental and assault GSWs is warranted.

With growing evidence for the negative mental health implications of GSWs for youth’s psychological functioning, interventionists have begun to identify how best to support patients by treating subclinical symptoms of psychological disorders following injury. Preliminary findings suggest the majority of pediatric patients (63%) do not receive any mental health services within the first six months following injury, including during their hospital admission [1, 2, 11]. Of the 37% who receive services, most are only offered during hospital admission where providers are focused on symptom evaluation. Thus, the primary goal of hospital-based psychological evaluations at present is stabilization and minimization of risk, meaning that treatment of subclinical symptoms is seldom offered [12]. Of the implemented interventions aimed at treating subclinical symptoms, providers typically focus on hospital-based violence intervention programs. Such interventions teach adaptive coping skills, including emotion regulation, and fostering healthy social supports in one’s community [13–15]. Through these programs, patients learn coping skills that can help counteract the emotional lability that often results from GSWs, while bolstering supportive social relationships which can protect against the development of mental health disorders later on [14]. Although studies have begun to highlight potentially useful interventions for youth who have suffered GSW injuries, a systematic review has yet to compile all known data and identify avenues for future research to aid in improving intervention efforts.

## Present study

**Fig. 1:**
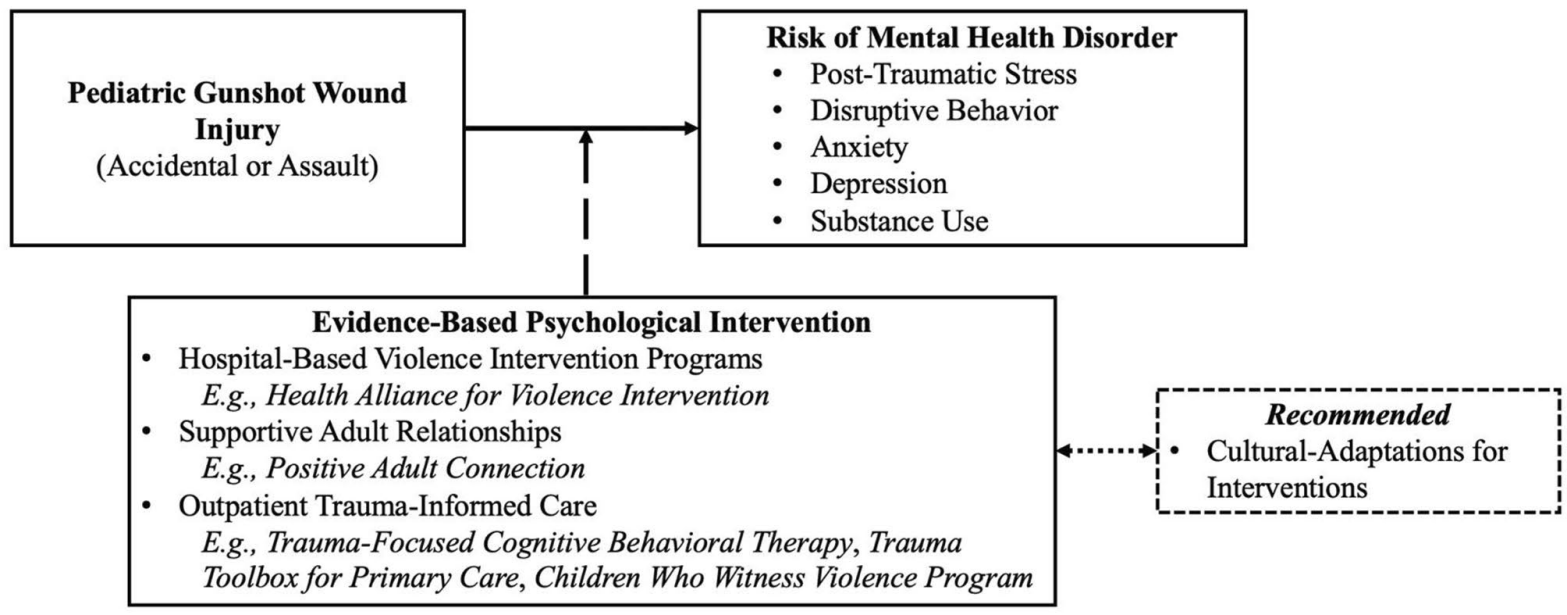
Conceptual Model: Hypothesized Relations between GSW, Mental Health Disorders, and Evidence-Based Psychological Interventions. Note. Unidirectional lines are supported by empirical evidence in the present review. Solid line represents a positive association, dashed line represents a buffering effect, bidirectional/dotted line is a suggested future direction based on data in the present review

To better understand the relation between GSWs and mental health outcomes for pediatric patients, the present study identified relevant articles published since 2018 following the modification to the Dickey Amendment. It was hypothesized that pediatric GSW patients would have significantly higher rates of mental health disorders when compared to non- and other-injured youth. A secondary goal was to synthesize data on evidence-based psychological interventions targeting subclinical symptoms of mental health disorders in this population. Interventions such as hospital-based violence intervention programs were hypothesized to moderate the relation between GSW injury and mental health outcomes, effectively treating subclinical psychological symptoms and relating to fewer long-term mental health disorder diagnoses when compared to untreated patients. To provide a visual aid regarding hypothesized relations, the present study proposed a conceptual model (Fig. 1).

## Method

### Search strategy

This systematic review was conducted in accordance with the Preferred Reporting Items for Systematic reviews and Meta-Analyses (PRISMA) 2020 guidelines [16]. The search began with a comprehensive review of the following medical and psychological databases: American Psychological Association (APA) PsycInfo, APA PsycArticles, Cumulative Index to Nursing and Allied Health Literature (CINAHL), Education Resource Information Center (ERIC), and Medical Literature Analysis and Retrieval Systems Online (MEDLINE). Relevant Boolean search terms were used to capture the following topics: pediatrics, guns/weapons, youth outcomes, and psychological interventions (Table 1). Following identification, all articles were extracted and deduplicated using validated procedures [17]. Article extraction and reviews were carried out using Rayyan software.

**Table 1:** Boolean search terms

Search Term Domain	Boolean Search Terms
Pediatric	Ped* OR child* OR infant OR baby OR adolescen* OR young adul* OR newborn OR toddler OR youth OR teen*
Gun/Weapon	gun* OR weapon* OR firearm
Youth Outcome	wound* OR trauma OR GSW OR spinal cord injur* OR SCI OR traumatic brain injur* OR TBI OR injur*
Psychological Intervention	psych* OR interv* OR therap* OR rehab* OR psychotherap* OR community heal* OR heal* center* eng*
Complete Boolean Search	(Ped* OR child* OR infant OR baby OR adolescen* OR young adul* OR newborn OR toddler OR youth OR teen*) **AND** (gun* OR weapon* OR firearm) **AND** (wound* OR trauma OR GSW OR spinal cord injur* OR SCI OR traumatic brain injur* OR TBI OR injur*) **AND** ( psych* OR interv* OR therap* OR rehab* OR psychotherap* OR community heal* OR heal* center* eng*)

Note. Search for the present review was completed on May 3rd, 2023

### Inclusion criteria and study coding

Identified articles were imported and screened by two independent reviewers. First, titles and abstracts for each article were reviewed using predetermined criteria: (1) peer-reviewed publication, commentaries, or theses/dissertations, (2) electronically published, (3) published in English, (4) published in or after 2018, (5) mean sample age from newborn to 20-years-old in line with the World Health Organization’s definition of a “youth,” (6) community sample, (7) no noted developmental disabilities prior to injury, (8) GSW victim, (9) accidental or assault as mechanism of injury, and (10) inclusion of mental health outcome and/or psychological intervention outcome following GSW injury. Given the increase in accidental and assault GSWs for youth [1], coupled with the correlation between premorbid mental health disorder and suicide attempts [18], articles exclusively reporting on self-injurious or suicide-related GSWs were excluded. The title and abstract for each paper were independently coded for inclusion/exclusion as 0 = *did not meet inclusion criteria* or 1 = *did meet inclusion criteria*. Following rankings, coders met to discuss discrepancies and resolve differences (Intraclass Correlation Coefficient; *ICC* = 0.95). Protocols regarding inclusion/exclusion criteria can be obtained via reasonable written request to the lead author (BLINDED FOR REVIEW).

### Full-text analyses, data extraction, and risk bias assessment

**Fig. 2:**
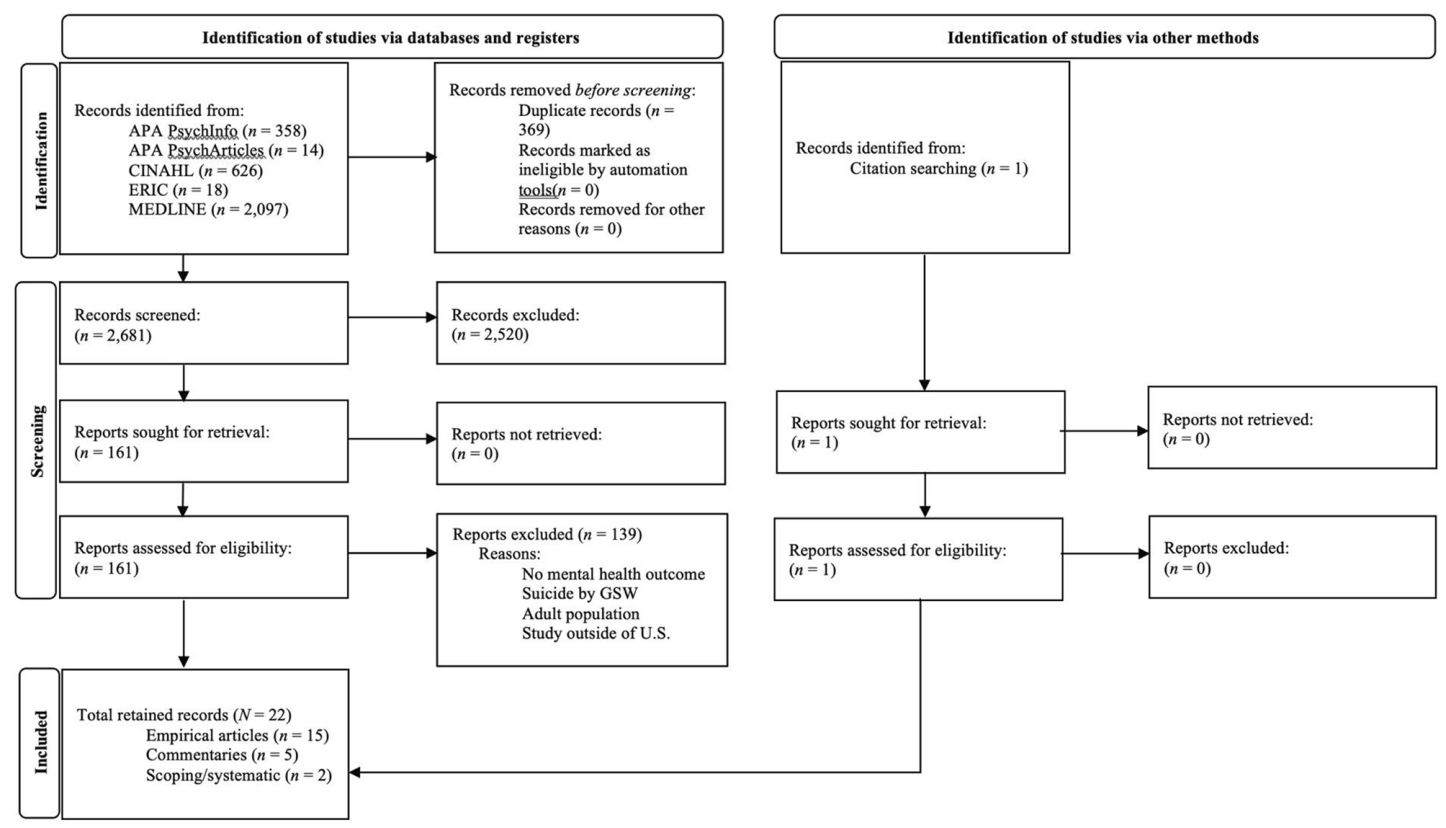
PRISMA 2020 flow diagram for new systematic reviews which included searches of databases, registers and other sources

Following title and abstract screenings, full-text reviews were conducted. Each review was again carried out by two independent coders and disagreements were resolved through discussion. Similar to the title and abstract review process, each article was coded as 0 = *did not meet inclusion criteria* or 1 = *did meet inclusion criteria*. A final sample of 22 articles was obtained (Fig. 2 for PRISMA flow chart) [17]. Once the full sample was identified, a risk bias assessment, Risk of Bias Assessment tool for Non-randomized Studies (RoBANS), was conducted for each empirical article (*n* = 15). Risk bias assessments were not conducted for commentaries in accordance with RoBANS guidelines [19]. Each empirical article was rated on six domains of potential risk as either *low*, *high*, or *unclear*. *Low* risk was found across all categories for all empirical articles and thus every identified article was retained for data extraction. For full details regarding RoBANS criteria, see Kim et al. (see Table 2 for RoBANS assessment by article) [19]. Data were then extracted following predetermined criteria (Table 3).

**Table 2:** Individual study risk bias assessment based on RoBANS criteria for empirical articles

Citation	1. Selection of Participants	2. Confounding Variables	3. Measurement of Exposure	4. Blinding of Outcome Assessments	5. Incomplete Outcome Data	6. Selective Outcome Reporting
Andrade et al., 2022	Low	Low	Low	Low	Low	Low
Bell et al., 2021	*NA*	*NA*	*NA*	*NA*	*NA*	*NA*
Bernardin et al., 2021	Low	Low	Low	Low	Low	Low
Borthwell et al., 2021	Low	Low	Low	Low	Low	Low
Cohen et al., 2021	Low	Low	Low	Low	Low	Low
Culyba et al., 2021	Low	Low	Low	Low	Low	Low
Cunningham et al., 2019(a)	Low	Low	Low	Low	Low	Low
Cunningham et al., 2019(b)	Low	Low	Low	Low	Low	Low
Ehrlich et al., 2022	Low	Low	Low	Low	Low	Low
Furman, 2018	*NA*	*NA*	*NA*	*NA*	*NA*	*NA*
Ganpo-Nikwenkwa et al., 2023	Low	Low	Low	Low	Low	Low
Ihle, 2021	Low	Low	Low	Low	Low	Low
Kuo et al., 2020	*NA*	*NA*	*NA*	*NA*	*NA*	*NA*
Lee et al., 2022	*NA*	*NA*	*NA*	*NA*	*NA*	*NA*
Magee et al., 2022	Low	Low	Low	Low	Low	Low
Manley et al., 2018	Low	Low	Low	Low	Low	Low
Mueller et al., 2022	Low	Low	Low	Low	Low	Low
Oddo et al., 2023	Low	Low	Low	Low	Low	Low
Phillips et al., 2020	Low	Low	Low	Low	Low	Low
Simpson et al., 2022	Low	Low	Low	Low	Low	Low
Thomas et al., 2021	*NA*	*NA*	*NA*	*NA*	*NA*	*NA*
Turner et al., 2021	Low	Low	Low	Low	Low	Low

**Table 3:** Gunshot wound mental health outcomes and evidence-based psychological interventions

Study (Type of Article)	Sample Size, Age, Gender, Race/Ethnicity	Site of Data Collection	Psychological Tool	Psychological Intervention Used/Recommended	Mental Health and Intervention Outcomes	Synthesis of Findings
**Andrade et al., 2022. (quantitative empirical article)**	*N =* 316 *M*_*age*_ = 16.1 years Gender = 92.4% male Race/Ethnicity = 84.1% Black	National Trauma Center Database	Psychological disorders noted in medical records.	*NA*	Comparing access to psychological supports for GSW patients in pediatric and adult centers by race.	On average, more Black youth were seen at adult centers when compared to pediatric centers and non-Black youth. Black youth were less likely to access psychological supports inpatient or outpatient given they were more likely to be treated at adult centers despite being pediatric patients.
**Bell et al., 2021. (commentary)**	*NA*	*NA*	*NA*	*NA*	Decreased parental supervision results in higher likelihood of suffering from a GSW. Pediatric GSW patients are at significant risk for developing psychological disorders which have negative implications for quality of life and overall functioning.	Author notes positive relation between decreased parental supervision and experiencing a GSW as well as risk for negative mental health and quality of life outcomes for pediatric patients.
**Bernardin et al., 2021. (quantitative empirical article)**	*N =* 407 *M*_*age*_ = 14.8 years Gender = 86% male Race/Ethnicity = 39% Black	St. Louis Children’s Hospital	Social work clinical interviews.	Hospital-Based Violence Intervention Program	Of the included participants, 37% met criteria for a current mental health disorder (details of disorder type not specified). There was no significant association between willingness to engage in a hospital-based violence intervention program and presence of a mental health disorder. Enrollment decreased readmittance to ED for violent injury from 11–4%.	While a large proportion of the sample had an active mental health diagnosis, the presence or absence of a mental health disorder did not significantly relate to patients’ willingness to enroll in the hospital’s violence intervention program. However, enrollment in the program did significantly decrease risk of recidivism to the ED for violent-related injuries suggesting positive outcomes for pediatric GSW patients who chose to enroll in the program.
**Borthwell et al., 2021. (quantitative empirical article)**	*N =* 115 *M*_*age*_ = 15 years Gender = 71% male Race/Ethnicity = 50% Black, 44% Latinx	UCLA Medical Center	Beside clinical interviews with psychologist or psychiatrist.	*NA*	Of the pediatric GSW patients, 43% of patients received a biopsychosocial assessment and 20% received a trauma evaluation. Of those who were evaluated, 70% were referred for trauma-related psychological services (i.e., some degree of traumatic response). Out of the entire sample, 23% were referred to child trauma clinic and 15% to hospital violence intervention program.	Of the patients included in the present study, less than half received some form of trauma evaluation in order to inform psychological referrals post-discharge. Of those who received an evaluation, almost three quarters were referred to some form of psychological, trauma-related outpatient services in order to cope with psychological challenges that resulted from injury.
**Cohen et al., 2021. (quantitative empirical article)**	*N =* 797 *M*_*age*_ = not provided (Range = < 12 years-old) Gender = 92.4% male Race/Ethnicity = 84.1% Black	Gun Violence Archive Data	Postulations implied from trends in publicly-available data.	*NA*	Pediatric GSW patients and exposure to gun violence was related to increased anxiety disorders and overall psychosocial stress.	Compared to prior to the COVID-19 pandemic, in the first 6-months children were more likely to experience a GSW. Of those who experienced a GSW, there was an increased risk for developing an anxiety disorder and overall psychosocial stress.
**Culyba et al., 2018. (quantitative empirical article)**	*N =* 143 *M*_*age*_ = 19.8 years Gender = 100% male Race/Ethnicity = 99% Black	Two Level 1 Trauma Centers in Philadelphia	Clinical interviews conducted by trained research coordinators.	Positive Adult Connection	In assessing the relation between adult-connection and GSW injury, 86% of patients stated they had a positive adult connection.	By having a positive adult connection, study hypothesizes that the risk for psychological challenges following a GSW in youth is decreased given the added social support such a relationship provides.
**Cunningham et al., 2019 (a). (qualitative empirical article)**	*NA*	Firearm Safety Among Children and Teens Consortium	Round robins for themes with pediatric and psychological experts regarding where to focus efforts to reduce GSW in children.	*NA*	Experts noted need to monitor for a variety of psychological outcomes in pediatric GSW patients including substance use and depression.	While PTSD is often screened for in pediatric GSW patients, experts note numerous other disorders may arise and should be closely monitored to help recommend and provide adequate psychological intervention when necessary.
**Cunningham et al., 2019 (b). (scoping review)**	*NA*	*NA*	*NA*	*NA*	Scoping review notes pediatric GSW victims are at higher risk for behavioral health challenges, especially PTSD, when compared to youth without a GSW.	The risk of significant psychological impairment, specifically PTSD, following a GSW is higher for youth when compared to youth who have not experienced the same type of injury.
**Ehrlich et al., 2022. (quantitative empirical article)**	*N =* 1,450 *M*_*age*_ = 15 years Gender = 83.4% male Race/Ethnicity = 64.7% Black	Medicaid MarketScan Database	Psychological disorders (ICD codes) noted in medical records.	*NA*	Compared to motor vehicle accidents, youth who experienced a GSW were at increased odds of developing a mental health disorder in the year following their accident. Specifically, 34.8% of GSW had a diagnosable mental health disorder a year later with 18.4% of patients who did not have a prior mental health disorder. Most common mental disorders were substance use, ADHD, trauma-related, conduct, and depressive disorders.	Pediatric patients who experienced a GSW were at increased risk of developing a mental disorder when compared to youth who had been in a motor vehicle accident. A call for the development of psychological interventions directly targeting GSW patients is proposed by the authors.
**Furman, 2018. (commentary)**	*NA*	*NA*	*NA*	Outpatient Trauma-Informed Care	Author proposes increased use of psychological interventions to combat psychological trauma from GSW in pediatric populations. Specifically, in pediatrician offices, “Trauma Toolbox for Primary Care” from American Academy of Pediatrics: for parents and professionals, website sources such as “Boston Medical Center’s Child Witness to Violence Project”), partner with “Children Who Witness Violence” programs.	The author calls for trauma-informed care of pediatric patients who have experienced a GSW in order to assist with negative mental health implications. Author provides specific recommendations to explore for pediatricians, parents, and mental health professionals.
**Ganpo-Nikwenkwa et al., 2023. (quantitative empirical article)**	*N = 24* (15 with GSW) *M*_*age*_ = 16.59 years Gender = 79.17% male Race/Ethnicity = 83.33% Black	Rochester Medical Center	Patient-Reported Outcomes Measurement Information System and Primary Care PTSD Screener from DSM-5.	*NA*	Of the 24 patients, 19 underwent psychological evaluations in hospital by psychiatry and were offered outpatient services. Two patients were evaluated by social workers and three received no evaluation. Eight patients followed-up with outpatient psychological services following discharge. Patients had significantly more substance use (45.8% vs. 13.0%) and symptoms of PTSD (41.7% vs. 6.8%) when compared to non-injured youth.	The majority of pediatric injuries were coupled with a psychological evaluation during inpatient stay. Compared to non-injured youth, pediatric patients, primarily due to GSW, were more likely to endorse lower overall mental health functioning including higher substance use and more PTSD symptoms. Findings underscore unique psychological challenges that occur in the wake of a GSW.
**Ihle, 2021. (systematic review)**	*NA*	*NA*	*NA*	Hospital-Based Violence Intervention Program	GSW is considered an ACE which results in increased risk for PTSD when compared to youth who have not experienced the same type of injury. Youth who engaged in hospital-based psychological intervention centered around posttraumatic growth were shown to report increased use of adaptive coping skills, emotion regulation, and report higher emotional well-being.	For children who engaged in psychological interventions following a GSW during their hospital stay, they were more likely to report adaptive psychological functioning and develop more adaptive coping skills compared to youth who did not engage in posttraumatic growth interventions.
**Kuo et al., 2020. (commentary)**	*NA*	*NA*	*NA*	Hospital-Based Violence Intervention Program	Author calls for more widespread implementation of hospital-based violence intervention programs focused on cultural competence to cope with pediatric GSW.	The author recommends taking a multidisciplinary approach, focusing on culturally-competent violence intervention programs in order to help pediatric patients cope with the negative mental health implications of GSWs as well as appropriately deliver therapy to youth from different backgrounds given most GSW victims come from under-resourced, racial minority backgrounds.
**Lee et al., 2022. (commentary)**	*NA*	*NA*	*NA*	Hospital-Based Violence Intervention Program	Given the noted increases in psychological disorders, broadly defined, in pediatric GSW victims, author calls for increased access and implementation of hospital-based violence intervention programs, noting that for GSW patients, enrollment in such programs reduces subsequent injuries by approximately 50% over the following 12 years.	The author notes the added risk for developing a psychological disorder in the wake of a GSW coupled with the decreased risk for re-injury when pediatric patients are enrolled in hospital-based violence intervention programs.
**Magee et al., 2022. (quantitative empirical article)**	*N =* 496 *M*_*age*_ = 16.1 years Gender = 74.0% male Race/Ethnicity = 76.5% Black	Indianapolis, Indiana	Medicaid claims (using primary and secondary diagnosis) - parsed apart into 3 categories: (1) stress/anxiety, (2) depression and mood, (3) disruptive behavior disorders.	*NA*	In the year following a GSW, patients obtained the following new diagnoses: substance use (12.2%), anxiety disorder (26.8%), depression (19.5%), and disruptive behavior (46.3%).	Pediatric GSW patients were diagnosed numerous diagnoses mental health conditions in the year following injury with the majority being disruptive behavior followed by anxiety, depression, and substance use disorders.
**Manley et al., 2018. (quantitative empirical article)**	*N =* 3,717 *M*_*age*_ = 14.0 years for White youth and 14.4 years for Black youth Gender = 84.2% male Race/Ethnicity = 67.0% Black	Nationally-Representative Database	Referral to mental health hospital following discharge as indicated in medical records.	*NA*	Of the youth with GSW injuries, White youth were more likely to be referred to a mental health hospital following discharge when compared to Black youth (1.3% vs. 0.1%).	White youth are more likely to be discharged to further psychological treatments indicating a disparity in access to mental health services following GSW for Black youth.
**Mueller et al., 2022. (qualitative empirical article)**	*N =* 316 *M*_*age*_ = 38 (health care providers) Gender = 92.4% male Race/Ethnicity = 84.1% Black	Twenty Site Database	Qualitative interviews with medical and mental health professionals regarding implementation of hospital-based violence intervention programs.	Hospital-Based Violence Intervention Program	Health professionals noted a significantly greater risk for reinjury and subsequent mental health disorder diagnoses following GSWs in youth when youth are not enrolled in hospital-based violence intervention programs. Interviews specifically highlight implementation of Life Outside of Violence Program.	Enrollment in hospital-based violence intervention programs supports youth by reducing risk of reinjury and mental health challenges following GSW.
**Oddo et al., 2023. (quantitative empirical article)**	*N =* 2,127 *M*_*age*_ = 13.5 years Gender = 81.0% male Race/Ethnicity = 48.0% Black, 22.0% White	National Medicaid Server	Medicaid claims regarding whether or not patient utilized mental health services following GSW.	Outpatient Trauma-Informed Care	Compared to non-injured youth, GSW patients were 1.40 times more likely to use mental health resources in the 12 months following their, 1.23 times more likely to use psychotherapy, and 1.40 times more likely to use substance use therapy.	GSW patients were more likely to engage in a variety of mental health services following injury when compared to non-injured youth.
**Phillips et al., 2020. (quantitative empirical article)**	*N =* 308 *M*_*age*_ = 14 years Gender = 87.0% male Race/Ethnicity = 54.8% White	Colorado	Medical records for mental health readmission 30 days and one year following GSW.	*NA*	Of all pediatric GSW patients, 14.3% followed-up with psychiatry for psychological challenges 30 days post injury. For adolescent GSW patients specifically, 13.7% followed-up with mental health 30 days following injury and all patients who were reevaluated 30 days post also followed-up one year after GSW.	There is a need to continue to triage for acute stress and post-traumatic stress symptoms during child and adolescent hospital stay given high rates of presenting mental health challenges and elevated number of psychological consultations following when compared to non-injured youth. Author notes need for greater use of hospital-based violence intervention programs in order to reduce risk of reinjury and mental health challenges.
**Simpson et al., 2022. (quantitative empirical article)**	*N =* 35,753 *M*_*age*_ = 15.47 years (non-readmission), 16.18 years (readmission) Gender = 87.02% male (non-readmission), 83.42% male (readmission) Race/Ethnicity = not provided	Nationwide Readmission Database	Mental health diagnoses as noted in medical records.	*NA*	Compared to non-readmission patients, pediatric GSW patients who had to go back to the hospital within 30 days of their injury due to medical reasons had significantly more anxiety (4.7% vs. 7.1%), mood (1.5% vs. 4.9%), bipolar (1.3% vs. 2.3%), psychotic (2.3% vs. 4.3%), alcohol use (2.3% vs. 4.3%), and drug use disorders (8.5% vs. 10.7%).	Pediatric GSW patients who required readmission to the hospital due to medical complications following their injury had significantly more mental health challenges compared to youth who did not require readmission.
**Thomas et al., 2021. (commentary)**	*NA*	*NA*	*NA*	*NA*	Author notes the elevations in rates of anxiety, depression, “chronic sorrow,” and post-traumatic stress disorder in pediatric GSW patients when compared to non-injured youth. Author also emphasizes demographic disparities such that Black males are more likely to fall victim to GSW when compared to any other demographic of youth.	Author notes mental health, racial, and gender differences in pediatric GSW patients with call for appropriate, responsive psychological interventions that help support these patients.
**Turner et al., 2021. (quantitative empirical article)**	*N =* 619 *M*_*age*_ = not provided (ranged from 2- to 17-years-old) Gender = 50.7% male Race/Ethnicity = 52.2% White, 45.2% Black	Boston, Philadelphia, rural Tennessee	Parent-report for youth ages 2- to 9-years-old: 26 items from the depression, anxiety, PTSD, and anger subscales on the from the Trauma Symptom Checklist for Young Children; self-report for 10- to 17-years-old: 24 items from the same subscales from the Trauma Symptom Checklist.	NA	Risk of GSW was higher for males, older children, youth living in urban areas, Black, non-Hispanic patients when compared to all other groups. Being a pediatric GSW patient was also associated with increased risk of PTSD symptoms when compared to youth who had exposure to gun violence but not experienced a GSW.	Gender, age, living conditions, and race/ethnicity were associated with risk of GSW. Additionally, obtaining a GSW injury was associated with higher risk of PTSD symptoms.

After data extraction and synthesis, a risk bias assessment for the present systematic review was conducted by two independent coders (Risk of Bias Assessment in Systematic reviews; RoBIS) [20]. Different from RoBANS, the RoBIS is used to analyze risk within a systematic review across a variety of domains: predetermined inclusion criteria, identification of relevant articles, data extraction, and synthesis of findings. Scores ranging from *low* to *high* risk were provided by independent coders, then an overall score was calculated. Risk of bias in the present review was deemed to be *low* across all domains (*ICC* = 0.97). All data analyzed during this study are included in the citations provided in the current manuscript.

### Inclusive language

Language in this systematic review is in accordance with the APA’s guidelines to prompt inclusivity and mitigate bias [21]. As such, some language from original articles has been modified in accordance with current APA standards (e.g., *White* instead of *Caucasian*). The shift in reported language was conducted to promote equity and inclusion in research and publication.

## Results

### Study descriptive and demographic differences

Prior to assessing rates of mental health disorders and evidence-based psychological interventions, sample demographic variables were assessed. Most empirical articles contained adolescent samples (age range = 13–20 years) [4, 5, 7, 10, 22–28]. Results suggest pediatric GSW patients were more likely to be Black, male, in mid-adolescence (i.e., ages 15–17 years old), living in an urban area, when compared to any other race, gender, age, and living environment [10, 29, 30]. Finally, Black youth were less likely to receive a psychological evaluation when compared to White youth as they were more likely to be admitted to an adult trauma center where psychological evaluations were less common when compared to pediatric hospitals [10]. 

### Mental health outcomes

Results suggested pediatric GSW patients were at higher risk for developing a mental health disorder, specifically post-traumatic stress, disruptive behavior, anxiety, depressive, and substance use disorders, when compared to non- and other-injured (e.g., motor vehicle collision; sporting accident) youth [4, 5, 7, 11, 22, 25, 28, 30, 31]. Studies varied regarding prevalence rates of diagnosed mental health disorders following GSWs; ranging from 1.5 to 46.3% of study samples [4, 22, 28]. One study reported that, despite the prominence of mental health concerns following a pediatric GSW, less than half of patients were triaged for symptoms of mental health disorders during hospital admission [23]. Further, two articles reported that, of the pediatric patients screened for psychological concerns, patients were typically only assessed for post-traumatic stress symptoms [7, 31]. 

### Evidence-based psychological interventions

The most frequently cited evidence-based psychological intervention for pediatric GSW patients following injury was hospital-based violence intervention programs (HBVIPs) [13–15, 22, 32]. Programs including *Health Alliance for Violence Intervention* [33], require an average of six sessions and focus on teaching adaptive coping skills (e.g., anger regulation, deep breathing, progressive muscle relaxation) and building supportive social relationships. HBVIPs identify the patient upon hospital admission, a period where patients are more likely to engage in psychological services due to ease of access compared to after discharge. Patients are connected with a mental health professional or trained “mentor” (i.e., adult from the community) who follow the patient during their hospital stay to assist in teaching curriculum-based coping skills. Studies suggested enrollment in HBVIPs minimized rates of GSW reinjury for pediatric patients from 11 to 4% when compared to youth who did not enroll. Such programs were also reported to significantly reduce subclinical symptoms of mental health disorders [14, 15, 22]. 

In addition to teaching coping skills, HBVIPs emphasize post-traumatic growth, defined as opportunities to create new meaning from a traumatic event [34]. Strategies utilized to promote post-traumatic growth include guided exposures in which the patient reimagines the traumatic event, putting words to what they were experiencing at the time of the event (e.g., describing what they saw, smelled, tasted, felt), expressing cognitions that occurred at the time and following the traumatic events, and reframing unhelpful cognitions that may center around concepts such as self-blame and guilt [22, 34]. Patients who engaged in HBVIPs reported increased subsequent use of adaptive coping and emotion regulation skills, as well as higher psychological well-being than youth who opted out of the program [13]. HBVIPs have been noted to be particularly useful for patients from marginalized backgrounds as access to mental health services are more easily obtained during hospital admission when compared to outpatient services following discharge [32]. Finally, HBVIPs allow for the integration of culturally-sensitive approaches, namely, discussion and implementation regarding how coping strategies and engagement with social supports may vary based on the social and cultural norms within the patient’s community [32]. 

A second identified psychological intervention was fostering and maintaining relationships with supportive adults in the patient’s community [24]. Similar to the community mentors utilized within HBVIPs, with the help of a mental health professional, this intervention, *Positive Adult Connection*, tasks pediatric patients with identifying “positive” adults in their life; someone with whom they feel close to, can discuss personal experiences with, and can seek for safety when they feel threatened or endangered. Supportive relationships can provide the patient with validation, emotional support, and problem-solving strategies after they are discharged from the hospital. Culbya and colleagues [24] reported of the pediatric GSW patients interviewed in their study, 86% reported they had at least one “positive adult connection.” Researchers posit such interpersonal relationships target subclinical symptoms, reducing the risk of developing subsequent mental health disorders. However, with only one study examining such effects, more evidence is needed to determine whether supportive adult relationships directly reduce mental health disorders post GSW injury.

The third proposed evidence-based psychological intervention was outpatient trauma-informed care, namely *Trauma-Focused Cognitive Behavioral Therapy*, *Trauma Toolbox for Primary Care*, and *Children Who Witness Violence Program* [27, 35–37]. Trauma-informed care is defined as “a treatment framework that acknowledges the effects of all types of trauma on [youth] and emphasizes physical and emotional safety during rebuilding to a sense of control and wellness.” [35, p. 2] Compared to non-injured youth, pediatric GSW patients are more likely to engage in outpatient trauma-informed care, likely due to post-traumatic symptoms that may result from an accidental or assault GSW [27, 35]. Treatments range in length from as short as a single session during a primary care visit to as long as 25 sessions with an outpatient therapist. Core to all trauma-informed interventions is a focus on validation of the patient’s emotional experiences and teaching adaptive coping skills [27, 35–37]. However, therapies varied in the degree of engagement and exposure the patient has with memories of their GSW. Evidence suggests exposure to traumatic events provides patients the opportunity to utilize newly learned adaptive coping skills when faced with intense emotional experiences while remembering the traumatic event. However, engagement in such therapies can be emotionally taxing on the patient, requiring youth to have the healthy social supports in place outside of therapy to help process the emotional turmoil that may follow intense therapeutic sessions [38, 39]. For a full list of synthesized results, see Table 3.

### Cultural adaptations for interventions

In light of the significant socioeconomic disparities regarding risk of pediatric GSW injury and access to subsequent mental health interventions [10, 26–28], Kuo and colleagues [32] recommended “culturally-competent violence intervention programs,” suggesting such modifications could drastically improve the mental health trajectories of pediatric patients from diverse backgrounds. Modifications could include an emphasis on understanding the importance of individual and community factors that contribute to the root cause of violence such as examining aspects of self-identity and cultural values around masculinity, strength, and the acceptability of support-seeking through involvement in mental health services. By acknowledging individual lived experiences and community stigmas, interventions are likely to increase patient engagement and decrease subclinical symptoms and long-term risk for mental health disorders.

## Discussion

Accidental and assault GSWs are the leading cause of death and the second-highest cause of injury for youth ages 1- to 17-years-old in the U.S [1]. For youth who survive an accidental or assault GSW, studies report up to half go on to develop a significant mental health disorder post-injury [1, 4, 5]. In light of the deleterious psychological implications of GSWs, the present study had two aims: (1) to synthesize known research published since 2018 regarding mental health outcomes for pediatric GSW patients and (2) identify evidence-based psychological interventions shown to treat subclinical symptoms of mental health disorders following a pediatric GSW injury. Hypotheses were supported and are depicted in the proposed conceptual model (Fig. 1).

### Mental health outcomes

Differing from self-injurious or suicide-related GSWs, accidental and assault GSWs do not necessarily imply the presence of a mental health disorder prior to the acquisition of injury [4, 5]. However, in support of Hypothesis 1, pediatric patients who have suffered an accidental or assault GSW are at significantly higher risk for developing subsequent mental health disorders when compared to non- and other-injured youth [4, 5, 7, 11, 22, 28, 30, 31, 40]. Elevations in rates of mental health disorders may be explained by the inherent traumatic nature of the injury. Such an event qualifies as a Criterion A stressor in the *Diagnostic and Statistical Manual of Mental Disorders (DSM)*, opening the door for a potential post-traumatic stress disorder diagnosis [21]. Additionally, other disorders including disruptive behavior and substance use are often diagnosed following an accidental or assault GSW [7, 31, 40]. These disorders may be brought on by maladaptive, avoidant coping in the aftermath of the traumatic event, particularly for youth who face difficulties with adaptive self-regulation. Avoidance can be expressed in a myriad of ways including use of substances to escape intense emotions or outward aggression/delinquency (i.e., disruptive behavior disorder) used to ignore heightened emotionality following trauma triggers (i.e., reminders of injury). If left untreated, avoidance can result in clinically significant mental health challenges [40]. Further, in the face of stress and avoidance, youth may develop exaggerated fear responses or feelings of hopelessness when adjusting to drastic changes to daily functioning (e.g., paralysis), giving way to anxiety and depressive disorders [5, 11]. Anxiety and depression are often comorbid, particularly during adolescence, a developmental stage that makes up the majority of GSW patients [4, 26, 41]. With increased risk for a myriad of mental health disorders, it is imperative that pediatric GSW patients be assessed for a wide range of psychological symptoms, even if symptoms have not yet crossed the threshold for clinical significance.

### Evidence-based psychological intervention outcomes

The second hypothesis that evidence-based psychological interventions would help in the treatment of subclinical symptoms of mental health disorders was supported. The most commonly cited type of intervention was HBVIPs including *Health Alliance for Violence Intervention* and was shown to be effective for numerous reasons [13, 14, 22, 32]. First, HBVIPs target accidental and assault GSW patients directly upon hospital admission, allowing for immediate access to services. HBVIPs typically only require a minimum of six sessions which can be completed during a hospital stay [22, 34]. Such immediate and efficient access to evidence-based interventions is crucial given that 63% of GSW patients do not receive psychological services within the first six months following injury [2]. Further, the majority of GSW patients are Black, adolescent males of whom data show are less likely to have access to mental health services following hospital discharge [10]. HBVIPs focus on building coping skills, creating and utilizing healthy relationships in one’s community, and engaging in post-traumatic growth [13–15, 22, 32]. By giving pediatric patients adaptive coping tools, HBVIPs empower patients to effectively cope with the intense emotionality that may follow injury. Incorporating HBVIPs as a standard of care for all pediatric GSW patients could be the first step in creating more equitable access to necessary mental health services following a GSW injury.

In addition to HBVIPs, Culbya and colleagues [24] detailed the value of identifying and building supportive adult relationships to minimizing mental health disorders following injury for pediatric GSW patients using the *Positive Adult Connection* intervention. Supportive relationships provide youth a person with whom they can process their emotions and problem-solve daily life challenges that result from significant injury. Supportive adult relationships can also help youth thrive after a GSW by serving as a checkpoint regarding their mental health and assist in identifying services if needed. Notably, despite research suggesting supportive adult relationships are advantageous for adaptive psychological functioning and minimizing mental health challenges during hospital stay [13–15, 22, 32], and valuable after hospital discharge [24], further research is needed to quantitatively test whether these relationships provide a statistically significant reduction in risk of mental health disorders when compared to youth without such supports.

Two studies noted trauma-informed care in an outpatient setting can be effective in buffering against symptoms of mental health disorders following a GSW [27, 35]. Treatments including *Trauma-Focused Cognitive Behavioral Therapy*, *Trauma Toolbox for Primary Care*, and *Children Who Witness Violence Program* assist patients in processing their injury and providing emotional support by reducing the potency of trauma triggers. Despite the potential of these psychological interventions for patients, significant barriers to access exist, particularly for youth from marginalized backgrounds. Barriers include, but are not limited to, cost, transportation challenges, limitations in trained providers, and lack of culturally-appropriate adaptations to interventions [42]. Additionally, it is important to note that not all pediatric GSW patients will develop significant mental health challenges after a GSW, nor will all patients with trauma symptoms be willing to engage in outpatient trauma-informed care. Considering individual differences and listening to patient preferences regarding engagement in psychological services following injury is crucial in supporting optimal long-term psychological functioning.

### Limitations

The present systematic review should be considered within the context of its limitations. First, included studies varied widely regarding study design and measures of mental health outcome data, barring meta-analyses. Given prior limited federal funding for GSW outcome research until 2018, studies included in the present review only span 2018 to present, truncating available data on pediatric GSW outcomes. Finally, no study quantitatively compared the effectiveness of psychological interventions for treating subclinical symptoms against one another. Future research may seek to empirically evaluate which interventions are most effective in treating subclinical symptoms in the wake of accidental or assault GSWs.

## Conclusions

In sum, youth who suffer an accidental or assault GSWs are at significantly higher risk for developing mental health disorders following injury when compared to any other form of pediatric injury. Despite this risk, many patients do not receive comprehensive evaluations of their symptoms or access to psychological services. Therefore, it is recommended that hospital-based mental health providers evaluate a broader range of symptoms to best identify patients’ mental health challenges and provide appropriate recommendations. Pertaining to evidence-based psychological interventions for treating subclinical symptoms, HBVIPs appear to be the most immediate and equitable interventions. Whether incorporating formalized programming into hospital settings, or adopting pieces of curricula within existing hospital structures, providers working with pediatric GSW patients may seek to employ aspects of these interventions to optimize psychological well-being of their patients. Finally, given the majority of pediatric GSW patients come from marginalized backgrounds [10, 29, 30], implementation of equitable, affordable mental health care following an accidental or assault GSW injury is paramount in reducing significant mental health disorders for *all* patients.

### Electronic supplementary material

Below is the link to the electronic supplementary material.

Supplementary Material 1

## Data Availability

All data analyzed in this study are included in the citations provided. Authors did not obtain additional data beyond that included in citations.

## Abbreviations

APA: American Psychological Association
CINAHL: The Cumulative Index to Nursing and Allied Health Literature
DSM: Diagnostic and Statistical Manual of Mental Disorders
ERIC: Education Resource Information Center
GSW: Gunshot Wounds
HBVIP: Hospital-Based Violence Intervention Programs
ICC: Intraclass Correlation Coefficient
MEDLINE: Medical Literature Analysis and Retrieval Systems Online
PRISMA: Preferred Reporting Items for Systematic reviews and Meta-Analyses
RoBANS: Risk of Bias Assessment tool for Non-randomized Studies
ROBIS: Risk of Bias Assessment in Systematic reviews
